# Post-colonoscopy Pseudotumour: A Rare Cause of Post-colonoscopy Acute Abdomen

**DOI:** 10.7759/cureus.99547

**Published:** 2025-12-18

**Authors:** Anushri Joshi, Muhammadhasan Anwaar, Boby Sebastian

**Affiliations:** 1 Medicine, West Suffolk Hospital, West Suffolk NHS Foundation Trust, Bury St Edmunds, GBR; 2 General Surgery, West Suffolk Hospital, West Suffolk NHS Foundation Trust, Bury St Edmunds, GBR; 3 Colorectal Surgery, West Suffolk Hospital, West Suffolk NHS Foundation Trust, Bury St Edmunds, GBR

**Keywords:** acute colonic haematoma, acute surgical abdomen, colonoscopy complications, ct use, intestinal pseudo-obstruction

## Abstract

A 41-year-old male, with a change in bowel habits and a positive faecal immunochemical test, underwent a routine colonoscopy without complications. The following day, he presented with an acute abdomen, with raised inflammatory markers and sepsis. Computed tomography of the abdomen and pelvis (CTAP) revealed stenosing lesions in the ascending colon and small bowel, with upstream dilatation. In view of the normal colonoscopy, we postulated that this could be due to bleeding into the colonic wall from the biopsies. A follow-up CTAP showed complete resolution of the masses with no further symptoms. Literature suggests that the likely cause was haemorrhage, leading to laminar mural thickening. Conservative management is usually sufficient for such cases. Correlating the clinical presentation, colonoscopy, pathology, and radiology results is important in the management of this rare complication. This case highlights a rare cause of acute abdomen. Awareness of such complications and correlating the various imaging modalities is crucial for patient management and avoiding unnecessary surgery.

## Introduction

Colonoscopy is a common tenet of diagnosis and treatment for several gastrointestinal conditions. It forms the backbone of the colorectal cancer screening programme, which is offered to a large proportion of the population based on age [1]. It is regarded as a relatively safe procedure, as reported complications are predominantly minor and transient. Serious post-procedural complications are uncommon and primarily associated with indications other than screening alone, e.g., polypectomy. Perforations, including those caused by therapeutic procedures, stand at a rate of <0.3% (in larger studies) to <0.1%. Haemorrhage, due to colonoscopy, is also reported to be around 0.1% to 0.6%, with the size of the polyp in a therapeutic procedure contributing to the bleeding risk [2,3].

We present a case of stenotic masses forming post colonoscopy in the ascending colon, resulting in symptoms of bowel obstruction, despite a complication-free procedure. This post-procedural complication is highly uncommon, with very few reported cases in existing literature. However, an awareness of this potentially life-threatening sequela will ensure early diagnosis for optimal patient management. Furthermore, by initially treating post-procedural complications as intervention-related, as done in this case, clinicians can more effectively identify the underlying cause of the presentation.

This case was presented at the ASGBI Conference on May 12th-14th, 2025 [4].

## Case presentation

A male in his 40s was referred for a colonoscopy through the straight-to-test (STT) pathway, a rapid access colorectal service. His eligibility was based on a change in bowel habits, abdominal bloating or discomfort, and a positive faecal immunochemical test (FIT) of 18.9 μg Hb/g. He had no relevant past surgical or medical history and was not on any long-term medications, except for awaiting a laparoscopic cholecystectomy for a gallbladder polyp.

**Figure 1:**
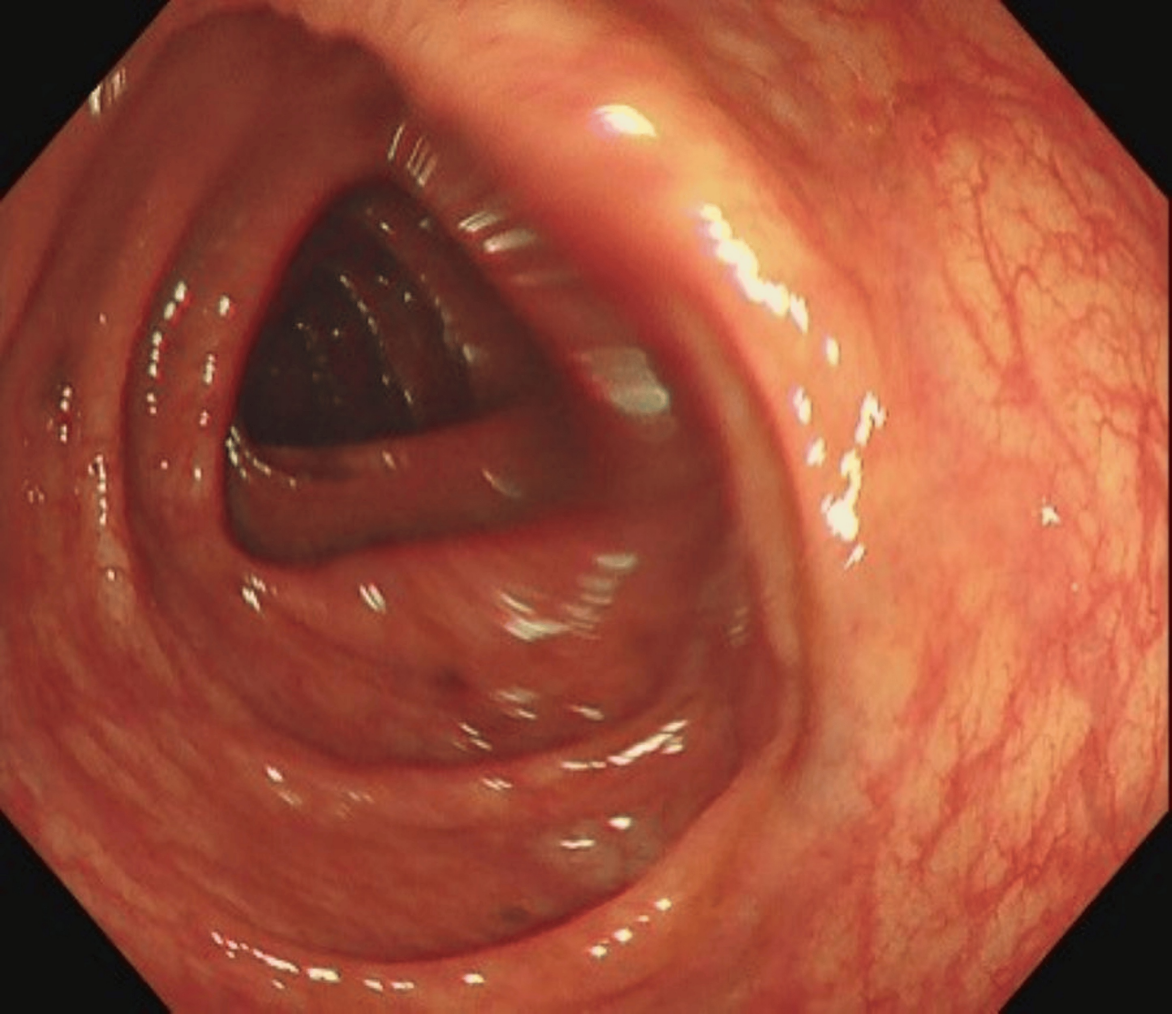
Ascending colon during colonoscopy. No abnormalities were seen.

The colonoscopy occurred without any complications. The biopsy was taken cold as per standard practice. There was no evidence of cancer within the lower GI tract, and several biopsies were taken from normal-looking mucosa in the right and left colon and the terminal ileum, as seen in Figure 1. The procedure was performed as an outpatient, and the patient was sent home with no complaints.

The following day, the patient presented to the emergency department with severe epigastric pain and an episode of vomiting and nausea. Examination revealed tachycardia, elevated respiratory rate, and a temperature of 39°C, with a mildly distended and rigid abdomen.

Investigations

Initial laboratory results showed leukocytosis (17.9 * 10^9^/l) and a CRP level of 13 mg/l. Haemoglobin and clotting studies were within normal range. Preliminary management consisted of intravenous fluids and antibiotics for the raised inflammatory markers.

**Figure 2:**
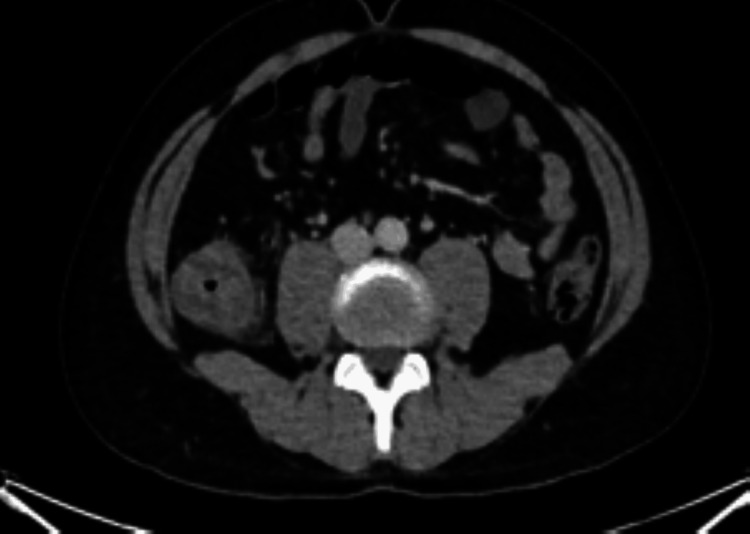
CT demonstrated a thickened ascending colon after the onset of obstructive symptoms.

An urgent computed tomography of the abdomen and pelvis (CTAP) was requested and revealed a stenosing lesion measuring 6.5 cm in the ascending colon, as seen in Figure 2. Perforation was excluded as a differential, as there was no free gas found.

Treatment

The patient was admitted, kept nil by mouth (NBM), and monitored for progression of his condition, whilst awaiting discussion with the colorectal multidisciplinary team (MDT). The worry was a missed tumour on colonoscopy, but the biopsies proved to be reassuring. Images of the biopsy samples are not routinely taken in the hospital. In view of the normal colonoscopy, we postulated that this could be due to bleeding into the colonic wall from the biopsies, resulting in laminar mural thickening. A trial of oral intake was started on the fourth day post colonoscopy. The patient was discharged on the sixth day post colonoscopy with an outpatient CT scan due to clinical improvement and laboratory results gradually normalising.

Outcome and follow-up

A follow-up outpatient CTAP showed complete resolution of the stenosing lesions that were previously present, alongside any evidence of bowel obstruction. He reported no further symptoms post discharge and, therefore, no further follow-up was needed.

Patient perspective

The patient stated that this investigation (colonoscopy) was a result of several months of gastrointestinal issues and blood found on the FIT test. He had no concerns prior to the procedure and felt that during the consent process, the risks were very well explained, although this particular risk (i.e., bleeding resulting in obstructive symptoms) was not mentioned. He felt that even if this was explained, it would not have affected his choice regarding having the colonoscopy. The colonoscopy occurred without any issues. In the evening, he started to feel severely unwell with fevers and abdominal pain associated with vomiting. In the morning, the general practitioner suggested he be taken to the accident and emergency (A&E) immediately due to his decreased level of consciousness and the severity of his symptoms. Following the CT scan, the general surgery team explained to him that there were suspicious lesions, but the colonoscopy was completely normal. He was in agreement with the surgical team regarding the watch-and-wait approach. He said that eventually he started feeling better. Eventually, he was discharged with no recurrence of any symptoms. He was left confused regarding the cause of his symptoms, but felt the care he received at the hospital was excellent, as there was no delay in scans or review from the surgical team. He agreed with the learning points of the case report and added that the responsiveness of the surgical and medical team reassured him that any serious pathology would be identified promptly. Although the experience was frightening, he stated that it had not deterred him from seeking medical care in the future. He hoped that sharing his experience would help improve patient understanding and preparedness for post-procedural complications.

## Discussion

This case highlights the significance of being aware of all potential complications of colonoscopy, regardless of their frequency in published literature. Abdominal pain is a commonly reported symptom post colonoscopy, but it can be attributed to several causes. Identifying the pathology responsible enables optimal patient management.

Colonoscopy is generally considered a safe procedure, with the risk of major complications such as perforation and haemorrhage being reported as less than 0.3% and 0.6%, respectively [2]. Additionally, these rates are affected by therapeutic interventions that may be carried out during the procedure, such as polypectomies. In line with standard hospital practice, the biopsy performed here was cold. This technique is associated with a lower risk of delayed bleeding and perforation from thermal injury compared to hot biopsy methods, such as those using diathermy. The majority of colonoscopies are surveillance only and, therefore, at a lower risk of these complications. Additional complications that have been reported, but with much lower incidence rates, include splenic injury, diverticulitis, bladder injury, mesenteric artery tear, and abdominal haematoma [5].

This case of a submucosal colonic lesion resulting in mechanical bowel obstruction is an extremely rare adverse event following a routine diagnostic colonoscopy. The most likely mechanism of formation of these lesions was due to haemorrhage, as evidenced by laminar mural thickening. An alternative explanation would be as a result of oedema, an even lesser reported complication. The literature review showed majority of the cases could be managed with conservative measures, such as the use of antibiotics and watchful waiting and would spontaneously resolve [6-8]. These patients were predominantly stable, with the main complaint being abdominal pain. Surgical intervention was offered in those cases where repeat CT scans (after the index scan for diagnosis) showed enlarging lesions and associated submucosal haematoma [9]. In this case, despite initial clinical instability, the patient’s obstructive symptoms began to rectify, and a repeat CT showed complete resolution of the stenotic lesions, which were ruled to be a haematoma.

In one of the cases where resolution was not spontaneously achieved, the patient needed an exploratory laparotomy with a right-sided hemicolectomy and an ileo-colonic side-to-side anastomosis. The extent of the hyperdense material on CT included a haematoma in the ascending colon but also extension into the peritoneal cavity, and perihepatic and perisplenic regions. There was also a retroperitoneal haemorrhage. There was a period of improvement following conservative management, but the patient quickly deteriorated, and a repeat CT showed an increase in the size of the lesions. After this, a decision to treat surgically was made [8].

This case describes obstructive symptoms occurring as a result of a likely colonic wall haematoma caused by submucosal bleeding, which was a diagnosis of exclusion as the initial CT was unable to distinguish further than a stenotic lesion. However, there are a couple of cases where obstructive symptoms are a result of internal hernias, entrapment of small intestinal loops in adhesions, ileus, and volvulus. This was more common in patients with previous bowel surgery resulting in adhesions [10-12].

All the reported cases make it abundantly clear that an index CT at the time of admission is the most effective way of differentiating between the serious complications of colonoscopies, determining the cause of obstructive symptoms and guiding management by establishing a baseline. They can diagnose perforation with more sensitivity than an erect chest X-ray and can also identify a potential bleed or a haematoma [13]. However, CT scanning is not always easily available because it is an expensive modality and poses radiation risks.

Given the potential for life-threatening deterioration of the patient’s presentation, ongoing review of both common and uncommon complications of colonoscopy presents an educational opportunity for clinicians involved in the procedure. It will likely aid in the identification of such complications and improve overall patient safety. Furthermore, following the approach taken here, considering post-procedural complications as intervention-related to begin with can also narrow down the diagnosis.

## Conclusions

This case provided an opportunity to observe a rare complication following an otherwise safe procedure. Future trainees should remain vigilant for both common and uncommon colonoscopy-related complications, employing early CT imaging when diagnostic uncertainty arises. By initially approaching post‑procedural complications as intervention‑related and carefully balancing conservative versus surgical management according to patient stability and imaging progression, overall patient safety can be enhanced.
